# Environmental exposures and the risk of multiple sclerosis investigated in a Norwegian case–control study

**DOI:** 10.1186/s12883-014-0196-x

**Published:** 2014-10-03

**Authors:** Marte Wendel Gustavsen, Christian Magnus Page, Stine Marit Moen, Anja Bjølgerud, Pål Berg-Hansen, Gro Owren Nygaard, Leiv Sandvik, Benedicte Alexandra Lie, Elisabeth Gulowsen Celius, Hanne F Harbo

**Affiliations:** Department of Neurology, Oslo University Hospital, Ullevål, Oslo, Norway; Institute of Clinical Medicine, University of Oslo, Oslo, Norway; Section of Epidemiology and Biostatistics, Oslo University Hospital, Ullevål, Oslo, Norway; Faculty of Dentistry, University of Oslo, Oslo, Norway; Department of Medical Genetics, University of Oslo and Oslo University Hospital, Oslo, Norway; Department of Immunology, Oslo University Hospital and University of Oslo, Oslo, Norway

**Keywords:** Multiple sclerosis, Environmental risk factors, Mononucleosis, Tobacco use, Hygiene hypothesis, HLA-DRB1*15:01

## Abstract

**Background:**

Several environmental exposures, including infection with Epstein-Barr virus, low levels of vitamin D and smoking are established risk factors for multiple sclerosis (MS). Also, high hygienic standard and infection with parasites have been proposed to influence MS risk. The aim of this study was to investigate the influence of various environmental exposures on MS risk in a Norwegian cohort, focusing on factors during childhood related to the hygiene hypothesis.

**Methods:**

A questionnaire concerning environmental exposures, lifestyle, demographics and comorbidity was administrated to 756 Norwegian MS patients and 1090 healthy controls. Logistic regression was used to calculate odds ratio (OR) with 95% confidence interval (CI) for the risk of MS associated with the variables infectious mononucleosis, severe infection during childhood, vaccination and animals in the household during childhood. Age, gender, HLA-DRB1*15:01, smoking and infectious mononucleosis were included as covariates. General environmental exposures, including tobacco use, were also evaluated.

**Results:**

Infectious mononucleosis was confirmed to be significantly associated with increased MS risk, also after adjusting for the covariates (OR = 1.79, 95% CI: 1.12-2.87, p = 0.016). The controls more often reported growing up with a cat and/or a dog in the household, and this was significant for ownership of cat also after adjusting for the covariates (OR = 0.56, 95% CI: 0.40-0.78, p = 0.001). More patients than controls reported smoking and fewer patients reported snuff use.

**Conclusions:**

In this Norwegian MS case–control study of environmental exposures, we replicate that infectious mononucleosis and smoking are associated with increased MS risk. Our data also indicate a protective effect on MS of exposure to cats during childhood, in accordance with the hypothesis that risk of autoimmune diseases like MS may increase with high hygienic standard.

## Background

Multiple sclerosis (MS) is an inflammatory disease of the central nervous system often affecting young adults [1]. Several environmental exposures have been associated with increased risk of developing MS, the best established are infection with Epstein-Barr virus (EBV), low levels of vitamin D and smoking [2]. The genetic background of MS is complex, and both HLA and non-HLA genes are known to influence the disease susceptibility [3]. HLA-DRB1*15:01 is the major risk allele for MS with an odds ratio (OR) of 3.1, whereas HLA-A*02:01 has been shown to have an independent protective effect with an OR of 0.73 [4]. Recently, 110 non-HLA MS susceptibility loci have been identified [4,5], all with relatively low ORs. The majority of the non-HLA loci are associated with immunologically relevant genes, supporting that MS primarily is an immune mediated disease.

Tobacco use and exposure have also been related to MS in several studies [2]. Smokers have an increased risk of MS, which also seems to increase with cumulative dose [6]. This association is not found for snuff use [6], which even has been suggested to have a protective effect on MS susceptibility [7]. Further, exposure to passive smoke has been suggested to negatively influence MS risk [8]. An interaction between smoking and the strongest genetic risk factors (carriage of HLA-DRB1*15 and absence of HLA-A*02) has been observed [9], indicating that this environmental exposure could have a stronger influence on genetically susceptible individuals.

EBV is a herpes virus infecting more than 90% of humans and the virus persists within the B lymphocytes after the infection [10]. A history of infectious mononucleosis (IM), the clinical manifestation of infection with EBV, is reported to give two to threefold increased risk of MS [11] and EBV seronegative individuals have a very low risk of MS [2]. Since infection with EBV in childhood often is asymptomatic, an episode of IM has been suggested as an indicator of low exposures to infections early in life [11]. Further, an interaction between antibodies against EBV nuclear antigen 1 and HLA-DRB1*15:01 status has been suggested [12,13].

MS affects more women than men, and the female to male ratio is increasing [14]. It has been hypothesized that oestrogen could have a protective role in MS [15] and an interaction between oestrogen and vitamin D has been suggested [16].

The prevalence of autoimmune diseases in general has increased in industrialized countries, and a reduction of exposure to pathogens early in life as a consequence of better hygienic standard has been proposed as a possible explanation for this, i.e. the hygiene hypothesis [17]. A relationship between MS risk and high sanitation standard was suggested as early as in the 1960s [18]. Parasite infections modulate the immune response in the host, and a possible protective effect of helminthic infections on disease progression in MS has been described [19]. Both cats and dogs are known hosts of parasites that can be transferred to humans [20], and households with animals have increased endotoxin levels in the house dust compared to households without animals [21]. Exposure to animals in relation to MS risk has previously been evaluated in some studies, but the findings are conflicting [22-26].

In this case–control study, we wanted to determine the frequency of various environmental exposures and lifestyle factors in a clinically and genetically well-characterized Norwegian MS cohort. We aimed both to replicate established risk factors and to investigate more recently suggested environmental exposures, with a main focus on exposures during childhood related to the hygiene hypothesis, such as severe infections, vaccinations and exposure to animals, hypothesizing that these exposures could influence inflammatory responses and thereby MS susceptibility.

## Methods

### Subjects and study design

MS patients (n = 756) from the Oslo MS Registry and randomly selected healthy controls (n = 1090) from the Norwegian Bone Marrow Donor Registry were invited in 2011/2012 to participate in this case–control study. The MS patients were diagnosed in accordance with the Poser and/or McDonald criteria [27,28]. The Oslo MS Registry and biobank has been built up over the last 24 years, and includes clinical data and DNA samples from a general, population based MS cohort [29], recruited from the largest MS clinic in Norway. The Norwegian Bone Marrow Donor Registry is a nationwide registry of blood donors who have consented to donate stem cells. Informed written consent was obtained from all the participants. The study was approved by the Regional Committee for Medical and Health Research Ethics South East.

A standardized questionnaire concerning lifestyle factors and background information was designed based on previously tested questionnaires [6,30,31]. This included questions about smoking, snuff use, exposure to passive smoke, animals in the household before the age of 18 (dog, cat, horse or other animals), IM, autoimmune comorbidity (type 1 diabetes, psoriasis, rheumatoid arthritis, ankylosing spondylitis, sarcoidosis, myasthenia gravis, celiac disease, inflammatory bowel disease, primary sclerosing cholangitis, systemic lupus erythematosus, Sjögren’s syndrome, hypothyroidism or hyperthyroidism), questions concerning female reproductive health, education level and current employment. In addition, a question concerning hospitalization for severe infection before the age of 18 (pneumonia, urinary tract infection, gastrointestinal infection, meningitis and/or encephalitis or other disease) was included. To evaluate the questionnaire, a test-retest was performed in 50 healthy subjects who were given the questionnaire twice with an interval of four weeks. The questionnaire was sent by mail to MS patients (n = 690) and controls (n = 1090) in 2011. Non responders received two reminders. In addition, 66 recently diagnosed MS patients were given the questionnaire directly when participating in another on-going study in 2012. The questionnaire was designed in the Cardiff TeleForm software and scanned in the same system. As quality control of the scanning process, 10% of the questionnaires were manually inspected.

### Genotyping

*HLA-DRB1* genotypes at four digit resolution were available for 319 patients and 908 controls, either obtained by a sequence based approach (n_ms_ = 75, n_controls_ = 908) [32] or imputed using HLA*IMP2 [33] for samples included in the Immunochip project [5] (n_ms_ = 244).

### Statistical analyses

Pearson’s Chi-square test and two sided t-test were used to analyse the personal and lifestyle related characteristics concerning tobacco use and exposure, comorbidity and female reproductive health. Logistic regression was used to calculate OR with 95% confidence interval (CI) for the risk of MS associated with the following exposure variables; IM, hospitalization for infection before the age of 18, participation in the childhood immunisation programme and animals in the household before the age of 18. Age, gender, HLA-DRB1*15:01 carrier status (present/not present), and the established environmental risk factors smoking (divided into ever smoker (daily and occasionally use combined) and never smoker) and episode with IM were included as covariates in multivariate logistic regression analyses. Information of vitamin D status was not available and could therefore not be adjusted for. Age was stratified into quartiles (<39, 39–45, 46–52 and > 52) to obtain linearity in the logit, and was thus analysed as a categorical variable. The exposure variables were in addition analysed in patients and controls < 60 years (n_ms_ = 398, n_controls_ = 914) with logistic regression adjusted for age (as a continuous variable), gender, smoking, IM and HLA-DRB1*15:01 status. Also, analyses of the significantly associated environmental exposures IM, ownership of cat or dog, smoking and snuff use were done in groups stratified on HLA-DRB1*15:01 carrier status. Analyses were conducted using IBM SPSS version 22 and a significance level of 5% was used for all analyses.

## Results

### Background characteristics

The total response rate was 70.1% (n = 530) in the MS patients and 84.2% (n = 918) in the controls. The demographic, genetic and clinical characteristics are presented in Table 1. There were more females among the patients (73.8%) than among the controls (58.3%), and the patient group had a higher mean age at inclusion in this study (50.5 vs. 44.0 years). The groups did not differ with regard to education level, but more controls than patients reported to currently be employed. The HLA-DRB1*15:01 allele was more frequently carried by the patients than the controls (58.3% vs. 29.0%, p_unadjusted_ = 8.03 × 10^−21^).

**Table 1:** **Background characteristics of patients and controls**

	**MS patients (n = 530)**	**Controls (n = 918)**
**Demographic characteristics**		
Female gender, n (%)	391 (73.8)	535 (58.3)
Age, mean (SD)	50.5 (12.7)	44.0 (6.9)
College/university, 4 years or more, n (%)	177 (33.6)	293 (32.5)
Nordic ethnicity, n (%)	487 (92.8)	877 (96.8)
Employed, n (%)	279 (52.8)	836 (95.7)
**Genetic characteristics**		
HLA-DRB1*15:01 positive, n (%)	186 (58.3)	263 (29.0)
**Clinical characteristics**		
Relapsing remitting onset, n (%)	454 (88.7)	-
Age at onset, mean (SD)	32.3 (9.3)	-
Disease duration, mean (SD)	18.2 (11.6)	-
Oligoclonal band status in cerebrospinal fluid positive, n (%)	448 (88.9)	-
EDSS, median (range)	3.0 (0–9)	-
MSSS, median (range)	3.25 (0–10)	-

*Abbreviations*: SD = Standard deviation, EDSS = Expanded Disability Status Scale, MSSS = Multiple Sclerosis Severity Score.

Missing values are not included in the calculations.

Table 2 presents personal and lifestyle related characteristics for patients and controls. Smoking was significantly associated with increased MS risk. Of the patients, 74.5% reported to ever having smoked compared to 54.8% of the controls (p_unadjusted_ = 1.15 × 10^−13^). Further, 11.4% of the patients and 15.6% of the controls reported to ever having used snuff (p_unadjusted_ = 0.029). No significant association to MS of exposure to passive smoke was seen. The groups did not differ with regard to autoimmune comorbidity, and no significant differences were detected with regard to tonsillectomy and appendectomy. Age at menarche and age when giving birth to their first child did not differ significantly between the female patients and controls, but the patients reported a significantly lower parity rate than the controls (1.21 vs. 1.77, p_unadjusted_ = 4.89 × 10^−14^).

**Table 2:** **Personal and lifestyle characteristics of included MS patients and controls**

	**MS patients (n = 530)**	**Controls (n = 918)**	**Crude p-value**
**Tobacco use and exposure**			
Ever smoker, n (%)	386 (74.5)	495 (54.8)	1.15×10^-13a^
Ever snuff use, n (%)	60 (11.4)	141 (15.6)	0.029^a^
Ever exposure to passive smoke, n (%)	380 (72.2)	652 (72.0)	0.94^a^
**Comorbidity**			
Autoimmune disease other than MS, n (%)	85 (16.0)	151 (16.4)	0.84^a^
Tonsillectomy, n (%)	118 (23.2)	174 (19.3)	0.078^a^
Appendectomy, n (%)	60 (11.8)	106 (11.8)	1.0^a^
Female reproductive health*			
Age at menarche, mean (SD)	13.07 (1.38)	12.97 (1.43)	0.28^b^
Number of children, mean (SD)	1.21 (1.07)	1.77 (1.12)	4.89×10^-14b^
Age at first child, mean (SD)	27.34 (5.50)	27.05 (4.79)	0.47^b^

*Abbreviation*: SD = Standard deviation.

^**a**^Pearson’s Chi square test.

^**b**^two sided t-test.

*Only women included (n_cases_ = 391, n_controls_ = 535).

Missing values are not included in the calculations.

### Environmental exposures during childhood and adolescence

Logistic regression analyses for environmental exposures during childhood and adolescence are presented in Table 3. A history of IM was significantly associated with increased MS risk, also after adjusting for age, gender, smoking and HLA-DRB1*15:01 status (OR = 1.79, 95% CI: 1.12-2.87, p = 0.016) when analysing all available data. Similar results were obtained when only including patients and controls < 60 years (OR = 1.88, 95% CI: 1.15-3.06, p = 0.012). The frequency of hospitalization for severe infection before age 18 did not differ between the groups, with exception of meningitis/encephalitis which was more often reported by the patients (1.9% vs. 0.3%, p_unadjusted_ = 0.007), but significance was not retrieved in the adjusted analysis. Participation in the childhood immunisation programme was equally high both for patients and controls (98.8% vs. 99.0%, p_unadjusted_ = 0.75). The patients less often reported to have had a cat or a dog in the household before age 18 compared to the healthy controls (39.7% vs. 51.0%, p_unadjusted_ = 3.6 × 10^−5^ and 36.9% vs. 47.1%, p_unadjusted_ = 1.73 × 10^−4^, respectively). This remained significant for ownership of cat after adjusting for the covariates (OR = 0.56, 95% CI: 0.40-0.78, p = 0.001), but significance for dog ownership was not retrieved in the adjusted analysis. When only including patients and controls < 60 years in this analysis, both cat and dog ownership were significantly associated in the unadjusted analysis (OR = 0.66, 95% CI: 0.52-0.85, p = 0.001, OR = 0.70, 95% CI: 0.55-0.90, p = 0.005, respectively), but only ownership of cat remained significant after adjusting for the covariates (OR = 0.59, 95% CI: 0.41-0.84, p = 0.004). The groups did not differ with regard to ownership of horse or other animals.

**Table 3:** **Environmental exposures during childhood and adolescence in patients and controls**

	**MS patients**	**Controls**	**Unadjusted analysis** ^**a**^	**Adjusted analysis** ^**b**^
	**(n = 530)**	**(n = 918)**	**p-value, OR (95% CI)**	**p-value, OR (95% CI)**
Infectious mononucleosis, n (%)*	84 (19.0)	106 (12.3)	0.001, 1.69 (1.23-2.30)	0.016, 1.79 (1.12-2.87)
**Hospitalized for infection before age 18, n (%)**				
Pneumonia	16 (3.1)	23 (2.5)	0.55, 1.22 (0.64-2.33)	0.67, 1.26 (0.45-3.56)
Urinary tract/kidney infection	16 (3.1)	25 (2.7)	0.73, 1.12 (0.59-2.12)	0.43, 0.64 (0.22-1.91)
Gastrointestinal infection	7 (1.3)	15 (1.6)	0.65, 0.81 (0.33-2.00)	0.36, 0.35 (0.038-3.28)
Encephalitis/meningitis	10 (1.9)	3 (0.3)	0.007, 5.91 (1.62-21.58)	0.76, 1.56 (0.95-23.37)
Other infection	27 (5.2)	37 (4.1)	0.33, 1.29 (0.78-2.14)	0.78, 1.10 (0.55-2.23)
**Participation in childhood immunisation programme, n (%)**	490 (98.8)	873 (99.0)	0.75, 0.84 (0.30-2.38)	0.88, 1.14 (0.21-6.20)
**Household pets before age 18, n (%)**				
Cat	207 (39.7)	463 (51.0)	3.6x10^−5^, 0.63 (0.51-0.79)	0.001, 0.56 (0.40-0.78)
Dog	192 (36.9)	427 (47.1)	1.73x10^−4^, 0.66 (0.53-0.82)	0.060, 0.73 (0.52-1.01)
Horse	32 (6.1)	63 (6.9)	0.54, 0.87 (0.56-1.35)	0.64, 0.86 (0.47-1.59)
Other animal	138 (26.5)	240 (26.5)	1.0, 1.0 (0.78-1.28)	0.85, 1.04 (0.71-1.52)

*Abbreviations*: SD = Standard deviation, OR = Odds ratio, CI = Confidence interval.

^a^Logistic regression, unadjusted.

^b^Logistic regression adjusted for age (stratified into following quartiles: < 39, 39–45, 46–52 and > 52), gender, smoking status, mononucleosis and HLA-DRB1*15:01 status.

*Not adjusted for mononucleosis.

Missing values are not included in the calculations.

### Analyses stratified on HLA-DRB1*15:01 carrier status

The environmental exposures which were significantly associated with MS in the unadjusted analyses (smoking, snuff use, IM and ownership of cat or dog before age 18) were also analysed with logistic regression adjusting for age, gender, IM and smoking in groups stratified on HLA-DRB1*15:01 carrier status (present/not present). All exposures showed significant association in MS patients carrying HLA-DRB1*15:01 allele, while only smoking was significantly associated in patients without this high risk HLA allele (Table 4). Importantly, the ORs and CIs overlapped between these two strata.

**Table 4:** Environmental exposures in patients and controls stratified on HLA-DRB1*15:01 carrier status

	HLA-DRB1*15:01 + (n_**MS**_ **= 186/n**_**C**_ **= 263)**	HLA-DRB1*15:01 - (n_**MS**_ **= 133/n**_**C**_ **= 645)**	HLA-DRB1*15:01 + (n_**MS**_ **= 186/n**_**C**_ **= 263)**	HLA-DRB1*15:01 - (n_**MS**_ **= 133/n**_**C**_ **= 645)**
	**Unadjusted analysis** ^**a**^ **p-value, OR (95% CI)**	**Unadjusted analysis** ^**a**^ **p-value, OR (95% CI)**	**Adjusted analysis** ^**b**^ **p-value, OR (95% CI)**	**Adjusted analysis** ^**b**^ **p-value, OR (95% CI)**
Smoking status*	4.0×10^−6^, 2.76 (1.79-4.24)	1.1×10^−5^, 2.62 (1.71-4.02)	2.02×10^−4^, 2.67 (1.59-4.48)	0.008, 2.01 (1.20-3.34)
**Snuff use**	0.006, 0.41 (0.22-0.77)	0.065, 0.55 (0.29-1.04)	0.20, 0.60 (0.27-1.32)	0.76, 0.88 (0.39-2.0)
Infectious mononucleosis**	0.25, 1.46 (0.77-2.77)	0.36, 1.29 (0.75-2.23)	0.033, 2.22 (1.07-4.61)	0.18, 1.55 (0.82-2.95)
**Cat in the household before age 18**	8.0×10^−5^, 0.46 (0.31-0.68)	0.062, 0.69 (0.47-1.02)	1.83×10^−4^, 0.41 (0.25-0.65)	0.26, 0.76 (0.48-1.22)
**Dog in the household before age 18**	0.013, 0.61 (0.42-0.90)	0.28, 0.81 (0.56-1.19)	0.052, 0.63 (0.39-1.01)	0.43, 0.83 (0.52-1.32)

*Abbreviations*: SD = Standard deviation, OR = Odds ratio, CI = Confidence interval, n_C_ = n_controls._

^a^Logistic regression, unadjusted.

^b^Logistic regression, adjusted for age, gender, smoking status and mononucleosis.

*Not adjusted for smoking status.

**Not adjusted for mononucleosis.

## Discussion

This study presents an extensive characterization of 530 Norwegian MS patients and 918 healthy controls with regard to environmental exposures, lifestyle factors and sociodemographic background. The observations confirm that IM and smoking are risk factors for MS also in a Norwegian cohort, and indicate that less studied environmental exposures, such as exposure to animals during childhood, could also influence MS susceptibility.

Environmental case–control studies are prone to diverse biases. In order to minimize a potential recall bias, the participants were not informed about the main hypotheses, and patients and controls received identical questionnaires. The selection of controls from the National Bone Marrow Donor Registry may have caused a selection bias towards a more than normally healthy control group. We do, however, note that educational level and comorbidity were similar in the patient and control groups, indicating that the groups are comparable with regard to socioeconomic status and thus probably also general health. Also, the percentage of present, daily smokers among the healthy controls was 16.7% (data not shown), which is equal to the frequency in the general Norwegian population [34], arguing for that the controls are representative with regard to lifestyle factors. Since the patients and controls as expected differed with regard to age and gender distribution, age and gender were included as covariates in the adjusted analyses. Moreover, information about established environmental and genetic risk factors for MS; smoking status, IM and HLA-DRB1*15:01, was available for a large fraction of the included individuals. Thus, we were able to adjust for these factors in the analyses, and also sub-analyses stratified for HLA-DRB1*15:01 carrier status could be conducted. Analyses for gene-environment interactions were not performed due to insufficient power. The analyses were not adjusted for vitamin D status and BMI, which could represent a limitation of the study. However, the relatively large number of participants and high response rates in both the patient and control groups are strengths of this study.

Smoking is an established risk factor for MS, but the mechanisms behind this association is still unclear [2,6]. The observations in the present study cohort, with a significantly higher proportion of the MS patients than controls reporting to be ever smokers, support that smoking increases MS risk. Further, in line with findings in a recent Swedish case–control study [7], we note that the patients less frequently reported snuff use. We found no significant association between the patients and controls for exposure to passive smoke, in contrast to some other reports [8].

Moreover, female reproductive factors have been investigated in relation to MS in several studies [35]. An early age at menarche has been suggested as a risk factor for MS [36], but the findings in the present study do not support this. We did, however, find a lower parity rate among the patients. It has previously been suggested that pregnancy and high parity could protect against MS [37,38], but the findings are conflicting [39]. Other factors, such as avoiding or postponing pregnancy due to concerns related to the disease or use of medications, must also be taken into consideration. In addition, medications used for MS could lead to reduced fertility [40].

In contrast to others [41,42], we found no differences between the patients and controls in the frequency of tonsillectomy, appendectomy or other autoimmune diseases than MS.

In this study, we observed that the controls more often were exposed to cats and/or dogs during childhood compared to the patients. Interestingly, when stratifying for HLA-DRB1*15:01 carrier status, exposure to cats was only significantly different in the HLA-DRB1*15:01 positive group. This trend was also seen for dog exposure. An association between infection with the parasite Toxoplasma gondii (T. gondii), (common in cats), and MS has recently been suggested [43]. T. gondii interacts with a number of genes in the host during an infection, and the study shows a large overlap of these genes with MS susceptibility genes. The biological implication of this observation is unclear. However, T. gondii is known to affect both pro-inflammatory and anti-inflammatory processes in the host [44], and this could theoretically influence MS susceptibility. Some previous studies have investigated exposures to animals in relation to MS risk, with conflicting findings [22-26]. In accordance with our observations, a protective effect of cat exposure on MS risk has been suggested in a Canadian case–control study including 200 MS patients and 202 controls, and this effect was even stronger in those who reported cat ownership for 10 years or more [24]. On the other hand, a positive correlation of MS risk and exposure to dogs has been suggested in some small studies [22,23]. Finally, others have not found an association between MS risk and exposure to household pets [25,26]. An Iranian case–control study of 394 MS patients and 394 controls did not detect any influence of exposure to animals on MS risk [25], and this was further supported by a German study among 245 MS patients and 296 controls [26]. The differences between the findings in the present study and these other studies could be due to better power in the present study. In addition, differences in the control groups among the German, Iranian and this Norwegian study may contribute to different results. Several aspects must be kept in mind regarding our observation of more exposure to cats and dogs in healthy controls than in MS patients. Presumably more outdoor activity and exercise in pet owners may reduce the risk of MS, by leading to higher levels of vitamin D and lower body weight. It should also be emphasized that a larger amount of the patients than the controls in the present study lived in or nearby Oslo, the capital of Norway, and this could be a potential bias when investigating ownership of household pets. Information of place of residency when growing up was not available in this study, thus the analyses could not be adjusted for level of urbanization. Notably, it has previously been shown that only 46.1% of the MS patients living in Oslo were born in Oslo [29].

Our findings add support to the suggested relationship between infection with EBV and MS [11,12]. Of the patients, 19.0% reported to have had IM compared to 12.3% of the healthy controls. An episode of IM could be an indicator of low exposures to infections early in life, since infection with EBV during childhood often is asymptomatic. The fact that EBV negative individuals (who may be assumed to have the highest level of hygiene) have very low MS risk could be considered as not in accordance with the hygiene hypothesis. However, an increased MS susceptibility due to few infections early in life could potentially be manifested only after infection with EBV [2].

Also, common childhood infections in relation to MS risk have been investigated in numerous studies [23-25,45], but severe infections during childhood are less studied. We did not detect any consistent significant differences, but an effect could have been missed due to modest power or recall bias. Further, both patients and controls reported similar participation in the childhood immunisation programme, supporting that childhood vaccinations do not increase the risk of MS, in accordance with previous findings [46].

## Conclusions

The observations in the present study both confirm the influence on MS risk of previously suggested environmental exposures such as smoking and IM and point to less established factors related to the hygiene hypothesis, like growing up with a cat in the household. The biological mechanisms behind these observations should be further investigated.
